# PD48 - Relationship between Second Hand Smoke (SHS) exposure and atopy in social disadvantaged asthmatic children

**DOI:** 10.1186/2045-7022-4-S1-P48

**Published:** 2014-02-28

**Authors:** Giuliana Ferrante, Velia Malizia, Maria Tornatore, Laura Montalbano, Roberta Antona, Giovanni Corsello, Stefania La Grutta

**Affiliations:** 1 Department of Health Promotion and Mother and Child, Università di Palermo, Palermo, Italy; 2 Institute of Biomedicine and Molecular Immunology-IBIM, National Research Council, Palermo, Italy

## 

The evidence of a relationship between second hand smoke (SHS) exposure and atopy is inadequate. Smoke habit prevalence is higher in lower parental educational levels. The aim of this study was to investigate the relationship between SHS and atopy in asthmatic children focusing on socioeconomic status (SES). We studied 170 outpatient asthmatic children with different levels of asthma (GINA guidelines). Medical history was taken in standardized way to determine prevalence of SHS exposure and maternal smoking during pregnancy. Information about the highest level of parental education was collected as a *proxy* of SES level. All patients underwent skin prick test (SPT) and spirometry according to international guidelines. Statistical analyses were performed using SPSS 19. 78 (45,9%) SHS exposed (SHSe) and 92 (54,1%) SHS non exposed (SHSne) asthmatic children were analyzed, aged 8.71±2.58 and 8.75±2.95, respectively. Exposure to maternal smoke in pregnancy was found only in SHSe (p <0.0001). With regard to SHSne, SHSe showed higher Body Mass Index (BMI) (19.50±4.17 *vs* 18.14±4.41, p <0.0044), higher percentage of the lower level of parental education (26.9% vs 13%, p <0.025). Moreover, SHSe showed a higher percentage of current exposure to pet (29.5% vs 16.3%, p <0.044) and at least one positive SPT, mainly indoor allergens (1.89 ±1.50 *vs* 1.45±1.39, p <0.062). No differences were found in pulmonary function tests (PFTs) and level of asthma even if SHSe showed more exacerbations than SHSne (3.19 ±4.23 *vs* 1.73±2.33, p <0.067). Exposure to SHS in children is associated with disadvantaged SES level and atopy.

